# Does Sestrin-1 Mitigate Cardiovascular Risks in Radiographic Axial Spondyloarthritis?

**DOI:** 10.5152/ArchRheumatol.2025.11138

**Published:** 2025-06-23

**Authors:** Selman Parlak, Ahmet Taha Sahin, Ahmet Lütfü Sertdemir, Adem Küçük, Abdullah İçli

**Affiliations:** 1 Department of Rheumatology, Beyhekim Training and Research Hospital, Konya, Türkiye; 2 Department of Cardiology, Beyhekim Training and Research Hospital, Konya, Türkiye; 3 Department of Cardiology, Necmettin Erbakan University Medicine Faculty, Konya, Türkiye; 4 Department of Rheumatology, Necmettin Erbakan University Medicine Faculty, Konya, Türkiye

**Keywords:** Cardiovascular involvement, CIMT, echocardiography, r-axSpA, Sestrin-1

## Abstract

**Background/Aims:**

Radiographic axial spondyloarthritis (r-axSpA) is a chronic inflammatory arthritis associated with an increased cardiovascular (CV) risk due to persistent inflammation. Sestrin-1, a stress-inducible protein with antioxidant and anti-inflammatory properties, has been implicated in cardiovascular protection. This study aimed to investigate the relationship between Sestrin-1 levels, cardiovascular markers, and echocardiographic findings in r-axSpA patients.

**Materials and Methods:**

This controlled study included 48 r-axSpA patients diagnosed according to the modified New York criteria and 48 age- and sex-matched healthy controls. Demographic, biochemical, and echocardiographic data were collected. Sestrin-1 levels were measured using an enzyme-linked immunosorbent assay kit, and carotid intima-media thickness (CIMT) was assessed via ultrasound. Statistical analyses evaluated differences between groups, as well as correlations between Sestrin-1 levels and inflammatory and cardiovascular parameters.

**Results:**

r-axSpA patients exhibited significantly lower Sestrin-1 levels compared to controls (*P* = .003). Sestrin-1 levels were negatively correlated with C-reactive protein (CRP) (r = −0.42) and erythrocyte sedimentation rate (ESR) (r = −0.38). Echocardiographic findings revealed increased CIMT (*P* < .001), reduced right ventricular systolic motion (RVSM), and lower tricuspid annular plane systolic excursion (TAPSE) in r-axSpA patients. No significant correlation was observed between Sestrin-1 levels and disease activity, as measured by the Ankylosing Spondylitis Disease Activity Score (ASDAS)-CRP.

**Conclusion:**

r-axSpA patients exhibit reduced Sestrin-1 levels and significant subclinical cardiovascular changes, including increased CIMT and impaired right ventricular function. These findings suggest that diminished Sestrin-1 may exacerbate oxidative stress and inflammation, thereby contributing to cardiovascular risk in r-axSpA. Further research is needed to explore the potential of Sestrin-1 as a biomarker for cardiovascular complications in r-axSpA.

**Main Points**

- Sestrin-1 levels were significantly lower in ankylosing spondylitis (AS) patients compared to healthy controls.

Reduced Sestrin-1 levels were negatively correlated with inflammatory markers CRP and ESR in AS patients.

AS patients exhibited increased carotid intima-media thickness and impaired right ventricular function on echocardiography.

No significant association was found between Sestrin-1 levels and disease activity scores, suggesting subclinical cardiovascular involvement.
The findings support a potential role for Sestrin-1 as a biomarker for cardiovascular risk in AS and warrant further longitudinal studies.

## Introduction

Radiographic axial spondyloarthritis (r-axSpA) is an inflammatory arthritis that predominantly affects the spine, often leading to ankylosis and typically manifesting as chronic back pain before the age of 45. The disease is frequently associated with peripheral joint and periarticular features, such as synovitis, enthesitis, and dactylitis. Moreover, r-axSpA can involve extra-articular manifestations, including uveitis, psoriasis, and inflammatory bowel disease. A substantial proportion of patients carry the human leukocyte antigen (HLA)-*B27* gene.^1^

Cardiovascular (CV) disease risk is increased in r-axSpA primarily due to chronic inflammation and traditional risk factors, with complications such as hypertension, heart failure, acute coronary syndrome, stroke, venous thromboembolism, conduction abnormalities, and occasionally, aortic root and valve disease contributing to CV morbidity and mortality.^2-4^

Sestrins are a stress-inducible protein family that regulate cellular metabolic networks, including the AMPK, mTORC1, and autophagy pathways.^5^ The tumor suppressor p53 directly stimulates the production of sestrin-1 and sestrin-2.^6^ Sestrin-1 exerts an antioxidant role by activating AMPK and inhibiting mTORC1 under conditions of DNA damage and oxidative stress.^7^ Emerging evidence underscores the critical importance of sestrins in maintaining cardiac homeostasis, particularly by preventing pathological cardiac hypertrophy through enhanced autophagy.^8^ Although numerous studies have investigated CV involvement in r-axSpA, the relationship between sestrin-1 and r-axSpA-associated CV pathology has not yet been explored. This study aims to evaluate the potential role of sestrin-1 in CV involvement in r-axSpA.

## Materials and Methods

This controlled cross-sectional study was conducted at Necmettin Erbakan University Hospital between July 2022 and June 2023. The study population included 48 patients diagnosed with r-axSpA based on the modified New York criteria and 48 age- and sex-matched healthy controls. The control group comprised asymptomatic individuals without any known chronic diseases or cardiovascular (CV) risk factors. The study was approved by the Institutional Review Board of Necmettin Erbakan University (Approval number: 2022/3896, date:22/07/2022), and all participants provided informed consent in accordance with the Declaration of Helsinki.

### Inclusion and Exclusion Criteria

r-axSpA patients aged 18-65 years with confirmed diagnoses and no history of CV events were eligible for inclusion. Exclusion criteria for all participants included hypertension, diabetes mellitus, dyslipidemia, renal failure, chronic heart or lung disease, and current use of CV medications. Additional exclusion criteria included pregnancy, smoking within the past year, and other systemic inflammatory diseases.

### Clinical and Laboratory Assessments

Demographic and clinical data, including age, sex, smoking status, and medication use, were recorded. Disease activity in r-axSpA patients was assessed using the Ankylosing Spondylitis Disease Activity Score (ASDAS). Venous blood samples were collected after 12 hours of fasting, centrifuged at 4000 rpm for 5 minutes, and stored at −80°C for subsequent analyses. Biochemical parameters, including lipid profile, liver and kidney function tests, C-reactive protein (CRP), and erythrocyte sedimentation rate (ESR), were measured using standard laboratory methods. Serum Sestrin-1 levels were quantified using a commercially available enzyme-linked immunosorbent assay (ELISA) kit (Human Sestrin-1 CD138 ELISA Kit, Bioassay Technology Laboratory, China) following the manufacturer’s protocol.

### Echocardiographic Evaluation

Comprehensive transthoracic echocardiography was performed for all participants by a cardiologist blinded to group allocation, using a Siemens Acuson S3000 ultrasound machine with a 2.5 MHz transducer. Measurements were conducted in accordance with the American Society of Echocardiography guidelines. The following parameters were evaluated:

- **Left ventricular (LV) dimensions**: End-diastolic (LVEDD) and end-systolic diameters (LVESD), interventricular septal thickness (IVST), and posterior wall thickness.
- **Right ventricular (RV) function**: Tricuspid annular plane systolic excursion (TAPSE) was measured using M-mode echocardiography, and right ventricular systolic motion (RVSM) was assessed qualitatively.
- **Left atrial (LA) dimensions**: Anteroposterior diameter was measured from the parasternal long-axis view.
- **Tricuspid regurgitation velocity (TRV)**: Evaluated using continuous-wave Doppler imaging.

### Carotid Intima-Media Thickness

Right carotid intima-media thickness (CIMT) was measured by an experienced cardiologist using the same ultrasound device, equipped with a 9L4 linear transducer (4.0-9.0 MHz). Measurements were obtained using B-mode imaging with participants in the supine position and the neck slightly extended. Carotid intima-media thickness was defined as the distance between the lumen-intima interface and the intima-media interface and was measured 3 cm proximal to the carotid bifurcation. To minimize variability, the mean of 3 consecutive measurements was recorded.

### Statistical Analysis

Statistical analyses were performed using R software version 4.4.2 (R Foundation; Vienna, Austria). Continuous variables were expressed as mean ± SD or median (interquartile range, [Q1–Q3]), while categorical variables were presented as frequencies and percentages. Group comparisons were conducted using Student’s *t*-test or Mann–Whitney *U* test for continuous variables, and the chi-square or Fisher’s exact test for categorical variables. Correlations between Sestrin-1 levels and echocardiographic, inflammatory, and biochemical parameters were analyzed using Spearman’s rank correlation coefficient. Statistical significance was set at *P* < .05.

## Results

Forty-eight axial spondyloarthritis (AS) patients and 48 healthy controls were included. The r-axSpA group consisted of 69% males (n = 33) and 31% females (n = 15), with a mean age of 40.13 ± 9.74 years and a mean ASDAS-CRP score of 2.9 ± 1.6. The median disease duration was 14 (7.75-20) years. In addition, the mean modified Stoke Ankylosing Spondylitis Spinal Score (mSASSS), assessed from radiographs obtained over the past 2 years, was 10 (5-18). Among r-axSpA patients, 35% (n = 17) were receiving sulfasalazine, while 65% (n = 31) were undergoing biological therapy (anti-TNF or anti-IL-17) (Table 1).

Table 2 presents demographic, electrocardiographic, echocardiographic, and laboratory parameters for both groups. Echocardiographic findings revealed reduced RVSM and TAPSE in r-axSpA patients compared to controls, along with increased LV end-diastolic diameter (LVEDD), LV end-systolic diameter (LVESD), LA diameter, and TRV (*P* < .001). Sestrin-1 levels were significantly lower in r-axSpA patients compared to controls (*P* = .003) with a negative correlation between Sestrin-1 levels and CRP/ESR.

## Discussion

In this study, evaluating the relationship between cardiovascular findings and Sestrin-1 levels in patients with r-axSpA, it was observed that r-axSpA patients had significantly lower Sestrin-1 levels compared to healthy controls. This reduction was associated with increased CIMT and subclinical right ventricular dysfunction, as evidenced by a decrease in TAPSE and RVSM. These findings suggest that reduced Sestrin-1 levels may exacerbate oxidative stress and inflammation, contributing to the cardiovascular burden associated with r-axSpA.

The relationship between Sestrin-1 and cardiovascular protection is supported by its established roles in mitigating oxidative stress and inflammation through the AMPK, mTORC1, and autophagy pathways.^8-10^ Previous studies have shown that Sestrin-1 reduces endothelial cell apoptosis and inflammation induced by oxidized low-density lipoproteins (ox-LDL), suggesting a protective role against atherosclerosis.^11^ In this study, the negative correlation between Sestrin-1 and inflammatory markers, such as CRP and ESR, further supports its potential role as an anti-inflammatory mediator in r-axSpA.

Increased CIMT, observed in the r-axSpA cohort, is a recognized marker of subclinical atherosclerosis and correlates with cardiovascular risk. Previous studies have similarly reported elevated CIMT in r-axSpA patients compared to healthy controls.^12,13^ Chronic systemic inflammation in r-axSpA, characterized by elevated pro-inflammatory cytokines such as TNF-α and IL-6, accelerates atherosclerosis by promoting endothelial dysfunction and vascular remodeling.^14^ The reduced Sestrin-1 levels in r-axSpA patients may further exacerbate these processes by impairing oxidative stress regulation and autophagic pathways, both of which are essential for vascular health.

Echocardiographic findings in this study revealed significant alterations in both right and left cardiac chambers in r-axSpA patients. The reduced TAPSE and RVSM suggest early right ventricular dysfunction, which may be associated with increased pulmonary vascular resistance or subclinical myocardial involvement due to chronic inflammation. Additionally, the observed increase in LV end-diastolic and end-systolic diameters, as well as left atrial size, is consistent with prior studies indicating diastolic dysfunction and subclinical myocardial remodeling in r-axSpA.^15^ The interplay between chronic inflammation, fibrosis, and microvascular dysfunction may underlie these echocardiographic abnormalities.

The absence of a significant correlation between Sestrin-1 levels and disease activity ASDAS-CRP in this study suggests that the cardiovascular effects of Sestrin-1 may be mediated independently of overt clinical symptoms. This finding is consistent with previous research indicating that subclinical cardiovascular changes in r-axSpA can occur even in patients with low disease activity.^2^ Moreover, it highlights the importance of assessing cardiovascular risk in all r-axSpA patients, regardless of symptom severity.

Emerging evidence suggests that interventions targeting oxidative stress and inflammation, such as TNF-α inhibitors, may improve vascular function in r-axSpA.^3^ The potential of Sestrin-1 as a therapeutic target warrants further exploration, given its ability to modulate key pathways involved in both inflammation and cardiovascular health. Preclinical studies have demonstrated that Sestrin-1 overexpression can attenuate pathological cardiac hypertrophy and improve vascular function.^8^ Translational studies are needed to determine whether enhancing Sestrin-1 activity could reduce cardiovascular risk in r-axSpA.

Despite these insights, the cross-sectional design of the study limits causal inferences regarding the relationship between Sestrin-1 and cardiovascular changes in r-axSpA. Longitudinal studies are needed to evaluate whether Sestrin-1 levels dynamically correlate with disease progression, cardiovascular events, or response to therapy. Additionally, the relatively small sample size and the lack of detailed data on participants’ lifestyle factors, such as physical activity and diet, represent limitations that should be addressed in future research.

## Limitations

This study has several limitations. First, the cross-sectional design precludes causal inferences regarding the relationship between Sestrin-1 levels and cardiovascular risk in r-axSpA. Longitudinal studies are needed to assess whether changes in Sestrin-1 levels over time correlate with clinical outcomes or disease progression.

Second, the relatively small sample size limits the generalizability of the findings. Larger cohorts with diverse demographics and comprehensive evaluations of additional cardiovascular markers could provide more robust evidence. Furthermore, the absence of detailed information on participants’ physical activity levels, dietary habits, and other lifestyle factors that may influence Sestrin-1 levels warrants further investigation.

This study demonstrates increased cardiovascular risk markers and decreased Sestrin-1 levels in r-axSpA patients. The findings highlight the potential cardioprotective role of Sestrin-1 and its possible utility as a therapeutic target in r-axSpA. However, larger prospective studies are needed to validate these hypotheses and establish Sestrin-1 as a clinically actionable biomarker for managing cardiovascular risks associated with r-axSpA.

## Figures and Tables

**Table 1.** Distribution of Demographic and Disease Characteristics

	**AS Patients (n = 48)**
Sex (Male); n (%)	33 (69)
Age (years); Mean±SD	40.13±9.74
Disease duration (years)	14 (7.75-20)
mSASSS	10 (5-18)
ASDAS-CRP; Mean±SD	2.9 ± 1.6
ESR, mm/hr.	12.85±10.64
CRP, mg/dL	10.7 ± 15.0
Treatment; n (%)* Sulfasalazin	17 (35)
Anti-TNF or Anti-IL 17	31 (65)

Anti-IL, anti-interleukin; AS, axial spondyloarthritis; ASDAS-CRP, Ankylosing Spondylitis Disease Activity Score–C-reactive protein; ESR, erythrocyte sedimentation rate; CRP, C-reactive protein; mSASSS, modified Stoke Ankylosing Spondylitis Spinal Score;

**Table 2.** Distribution of Data According to Patient and Control Groups

	Control Group (n = 48)	AS Patients (n = 48)	*P*
Sex (Male);n(%)	31 (56.4)	33 (69)	** .026**
Age (years)	57.56 ± 10.47	40.13 ± 9.74	.388
PR	145.02 ± 19.83	135.52 ± 11.45	.002
QRS	84.78 ± 12.61	87.15 ± 7.90	** .201**
QTC	376.27 ± 33.41	390.88 ± 36.42	** .034**
LVEDD (mm)	46.48 ± 3.45	45.6 ± 0.34	**<.001**
LVESD (mm)	27.87 ± 3.55	28.4 ± 0.4	**<.001**
LA (mm)	34.69 ± 5.47	32.3 ± 0.37	**<.001**
Mitral E (cm/s)	59.09 ± 8.30	73.07 ± 17.29	**<.001**
Mitral A (cm/s)	46.09 ± 6.88	63.56 ± 14.89	**<.001**
RV SM	14.74 ± 1.20	12.84 ± 1.67	**<.001**
TAPSE	2.83 ± 0.19 (2.7-3.0)	2.46 ± 0.38 (2.1-2.8)	**<.001**
CIMT-right(mm)	0.56 ± 0.07 (0.5-0.6)	0.61 ± 0.12 (0.5-0.7)	** .036**
CIMT-left (mm)	0.56 ± 0.09	0.61 ± 0.15	.064
Sestrin-1 levels	23.22 ± 18.68	15.62 ± 4.72	.003

AS, axial spondyloarthritis; CIMT, carotid intima-media thickness; LVEDD, left ventricular end-diastolic diameter; LVESD, left ventricular end-systolic diameter; LA, left atrial; TAPSE, tricuspid annular plane systolic excursion.

## Data Availability

The data that support the findings of this study are available upon request from the corresponding author.
